# Burden and Expressed Emotion of Caregivers in Cases of Adult Substance Use Disorder with and Without Attention Deficit/Hyperactivity Disorder or Autism Spectrum Disorder

**DOI:** 10.1007/s11469-015-9567-9

**Published:** 2015-07-21

**Authors:** Linda M. Kronenberg, Peter J. J. Goossens, Jooske T. van Busschbach, Theo van Achterberg, Wim van den Brink

**Affiliations:** Department of Residency Training MANP Mental Health, Dimence, Deventer, The Netherlands; Expertise Centre Developmental Disorders, Dimence, Deventer, The Netherlands; GGZVS, Institute for the Education of Clinical Nurse Specialists in Mental Health, Utrecht, The Netherlands; SCBS, Dimence, Deventer, The Netherlands; Scientific Institute for Quality of Healthcare, Radboud University Nijmegen Medical Centre, Nijmegen, The Netherlands; University of Groningen, University Medical Centre Groningen, Groningen, The Netherlands; Centre for Health Services and Nursing Research, KU Leuven, Leuven, Belgium; Department of Public Health and Caring Sciences, Uppsala University, Uppsala, Sweden; Amsterdam Institute for Addiction Research, Academic Medical Center University of Amsterdam, Amsterdam, The Netherlands; Dimence, P.O. Box 5003, 7400 GC Deventer, The Netherlands; Department of Public Health, University Centre for Nursing and Midwifery, Faculty of Medicine and Health Sciences, Ghent University, Ghent, Belgium

**Keywords:** Burden, Expressed emotions, Substance use disorder, Attention deficit/hyperactivity disorder, Autism spectrum disorder

## Abstract

**Objective:**

To identify and compare caregiver burden and expressed emotion (EE) in adult substance use disorder (SUD) patients with and without co-occurring attention deficit/hyperactivity disorder (ADHD) or autism spectrum disorder (ASD). To examine possible differences in correlations between caregiver burden and EE across patient groups.

**Design and Methods:**

Cross-sectional study with measures of perceived burden (Involvement Evaluation Questionnaire: IEQ), subjective stress (General Health Questionnaire: GHQ) and perceptions of expressed emotion (Level of Expressed Emotion: LEE) in informal caregivers for patients with SUD, SUD+ADHD or SUD+ASD.

**Findings:**

No differences in caregiver burden or expressed emotion when caregivers for patients with SUD were compared to caregivers for patients with SUD+ADHD. A moderate but non-significant difference for caregivers of patients with SUD versus SUD+ASD, which disappeared when the number of contact hours between patient and caregiver for the SUD only group was controlled for. The IEQ sum scores also substantially correlated with the LEE sum scores.

**Conclusion:**

Informal caregivers for patients with only SUD show higher levels of burden and EE than informal caregivers for patients with SUD and a co-occurring ASD. This difference was largely explained by the higher number of contact hours between patient and caregiver in the SUD only group.

## Background and Review of the Literature

Since the 1950s, it has been acknowledged that informal care for the mentally ill may result in psychological problems on the part of caregivers (Platt 1985; Yarrow et al. 1955). Goossens et al. (2008) define an *informal caregiver* as *“the person who, in the perception of the patient, is an important person in his or her life, who is not a professional and who delivers significant support and care for the patient”*. In this article, this is what we will refer to as a “caregiver”. In contrast, a professional caregiver will be referred to as “clinician”.

Caregivers are important partners for clinicians in the care for patients with psychiatric disorders. Caregivers can provide companionship and emotional support for patients but also assist with their personal care, household tasks, financial tasks and the management of medication. Caregivers may also communicate with health professionals at times and serve as an advocate for the patient during medical appointments and hospitalization. Caregivers can also play a key role as the informal care coordinator for a patient (Feinberg et al. 2011).

Given the importance of caregivers, Schulze and Rossler (2005) concluded that effective mental health service delivery should, among other things, focus on the input of caregivers’ resources and competences. Therefore caregivers should be considered being expert partners in psychiatric practice and research. As Riebschleger et al. (2008) later concluded, however, medical guidelines and the medical education curriculum do not pay sufficient attention to the role of families in the care for people with mental illness.

Studies show that the *burden* on informal caregivers for the mentally ill is substantial (Caqueo-Urizar and Gutiérrez-Maldonado 2006; Fadden et al. 1987; Gutiérrez-Maldonado et al. 2005; Hatfield and Lefley 1987; Schene et al. 1994; Van Wijngaarden 2003). Burden is the personal suffering of a caregiver as a consequence of the illness of a family member or friend. Caregiver burden can include grief; loss of hope, dreams and expectations, and even despair in cases of relapse. Caregiver burden can also refer to the problems and challenges confronting caregivers such as the symptoms of the patient’s illness, disturbed (family) relationships, limitations of the professional health care system, social stigma, economic/financial losses, decreased health of other family members and decreased social networks and support (Schene 1990). Many caregivers report experiencing feelings of loss but also anxiety and depression (Fadden et al. 1987). If the experienced burden exceeds the supportive powers of a caregiver, moreover, they themselves may develop mental health problems as well (Barrowclough et al. 2005; Kwekkeboom 2000; Möller–Leimkühler 2005; Van Wijngaarden 2003; Van Wijngaarden et al. 2009).

With regard to the *patients* receiving the help of informal caregivers, Vaughn and Leff (1981, 1985) found that patients with schizophrenia needed more relapse care when they returned from inpatient treatment to a home environment with high levels of negative expressed emotion (EE) as opposed to low levels of such EE on the part of caregivers. Vaughn and Leff further identified four typical characteristic attitudes or response styles which tend to distinguish relatives who are highly critical or overinvolved from those who are not, as judged by their reported behaviour toward the patient : (1) emotional response, (2) attitude, (3) level of tolerance and expectations, and (4) level of intrusion in the patient’s life.

EE is important for understanding and preventing relapse among the mentally ill (Butzlaff and Hooley 1998). Informal care can bring benefits but also risks for patients—depending on—among other things—the degree of negative EE. Very little is known about caregiver burden in relation to EE and, for which matter, caregiver burden in relation to specific psychiatric disorders.

According to epidemiological surveys, the global lifetime prevalence of SUD in the general population is between 10 and 20 % (Jacobi et al. 2004; Kessler et al. 2005) and about two-thirds of SUD patients are dual diagnosis patients (Chan et al. 2008). In this current study, the focus is on the dual diagnosis of SUD with Attention Deficit/Hyperactivity Disorder (ADHD) or Autism Spectrum disorder (ASD). In a recent meta-analysis of patients with a SUD, 25 % also met criteria for adult ADHD (van Emmerik-van Oortmerssen et al. 2012) while the lifetime prevalence of SUD in patients with ASD has been reported to range from 11 to 29 % (Hofvander et al. 2009; Lugnegard et al. 2011; Singh et al. 2012; Sizoo et al. 2010). A recent study by Smith et al. (2014) further reports that a gene linked to ASD has also been found to play a critical role in the incidence of addiction-related behaviours.

In a study by Heflinger and Brannan (2006), similar levels and types of caregiver strain are reported for those families caring for youth with SUD and those families caring for youth with other mental health problems. However, Cleary et al. (2008) found the caregivers for patients with psychiatric disorders and co-occurring SUD to report significantly higher levels of anxiety than the caregivers for patients with psychiatric disorders but no SUD. When Werner and Shulman (2013) recently studied the subjective well-being of caregivers for patients with developmental disabilities, moreover, they found their subjective well-being to be below the normative score for Western populations and the subjective well-being of those caring for individuals with ASD to be particularly low. In a recent study of caregiver burden in relation to patients with ASD or ADHD transitioning into adolescence and adulthood Cadman et al. (2012) found both disorders to be associated with very high levels of caregiver burden.

According to Cadman and colleagues, ASD leads to even more caregiver burden than ADHD. In both groups, caregiver burden is mainly explained by the unmet needs of the patient (i.e., depression/anxiety and inappropriate behaviour). For adolescents in the same study with ASD only, significant associations between burden and unmet needs are reported for such domains as social relationships and major mental health problems. Cadman et al. (2012) further conclude that the level of burden for the caregivers of patients with ASD is comparable to that reported for the caregivers of patients with brain injury. In the recent study by Kronenberg et al. (2014), SUD-only patients were further shown to have fewer care needs than SUD patients with co-occurring ADHD or ASD. And the SUD patients with co-occurring ASD showed the greatest number of care needs and more extensive care needs than the patients with SUD only and the patients with SUD and co-occurring ADHD.

In sum, informal caregivers have an important role to play in the care for patients with a mental illness. In addition to providing support but also companionship and guidance, they can also play a role in relapse prevention and thus help reduce care costs. The burden of caring for patients with ASD or ADHD is known to be relatively high. In addition, the caregivers for patients with a psychiatric disorder and a co-occurring SUD have been found to report significantly higher anxiety levels than the caregivers of patients without a co-occurring SUD. To our knowledge, however, the burden of caring for adult SUD patients either with or without a co-occurring ADHD or ASD has yet to be systematically investigated. These caregivers are nevertheless at a major risk of developing mental health problems as our review of the relevant studies has revealed. And we therefore conducted the study reported on here in order to answer the following research questions.

1. What is the relative burden of caregivers of adult SUD patients with or without a co-occurring ADHD or ASD? (*alternative hypothesis*: *Co-occurrence of ADHD or ASD does not raise burden of care-givers of patients with SUD*.)
2. What is the relative perceived EE of caregivers of adult SUD patients with or without a co-occurring ADHD or ASD according to caregivers themselves and patients? (*alternative hypothesis*: C*o-occurrence of ADHD or ASD does not raise level of EE for caregivers of patients with SUD, from either the perspective of the caregiver or the patient*.)
3. Does perceived caregiver EE from the patient’s perspective versus the caregiver’s perspective differ for the three groups of patients? *(alternative hypothesis: No significant differences in perceptions of caregiver EE across three patient groups when viewed from the perspectives of the caregiver* versus *the patient.*)
4. Does perceived caregiver EE correlate with caregiver burden in the three groups of patients? *(alternative hypothesis: No significant correlations between caregiver EE and caregiver burden for different groups of patients.*)

## Methods

### Study Design

A cross-sectional study was conducted with a population of caregivers and treatment-seeking SUD patients who either had a comorbid diagnosis of ADHD, ASD or not such comorbid diagnosis.

The study was approved by the medical ethics committee and by the institutional review boards from all participating institutions. All patients and their caregivers received both verbal and written information about the study. And the patients and caregivers signed an informed consent form prior to the start of the study.

### Participants

The target population consisted of SUD patients either with or without a comorbid ADHD or ASD and their caregivers. Participation of patients in the study was based upon the following inclusion and exclusion criteria. Inclusion: outpatient treatment for SUD; age 18–65; IQ >80; current DSM-IV diagnosis of SUD either with or without current DSM-IV diagnosis of ADHD or ASD; and mastery of the Dutch language. Exclusion: diagnosis of SUD with both comorbid ADHD *and* comorbid ASD. All patients were recruited from mental health services and addiction treatment services which routinely conduct clinical screening and diagnostic procedures. The present study follows up on a study of 122 patients by Kronenberg et al. (2014). The same 122 patients were asked to participate in the present study. In most cases, the patients who had agreed to participate asked their own caregivers to participate in the study as well. In some cases, with the permission of the patient, the researchers asked the caregivers directly to participate in the study.

### Data Collection

To measure caregiver burden, the Involvement Evaluation Questionnaire (IEQ: van Wijngaarden and Schene 1997) was administered. The IEQ consists of 81 questions which can be classified into seven modules covering the past 4 weeks. The first module, items 1–15, address socio-demographic information. The second module, items 16–46, constitutes the core module of the IEQ and addresses the consequences of a patient’s psychiatric disorder for the caregiver. The items address the encouragement and care provided by the caregiver; watching for dangerous patient behaviour; interpersonal problems between the patient and caregiver; caregiver worries; and caregiver coping and expected burden. All of the items are scored along a 5-point Likert scale (0 = never, 1 = sometimes, 2 = regularly, 3 = often, 4 = always). The third module, items 47–54, assesses the financial burden for the caregiver due to the patient’s illness. The fourth module, items 55–66, calls upon the questions from the General Health Questionnaire (GHQ; Goldberg and Williams 1988) to assess the mental and somatic burden for the caregiver and caregiver distress. All of the items in this module are responded to along a 4-point Likert scale (0 = better than usual, 1 = the same as usual, 2 = worse than usual, 3 = much worse than usual). The fifth module, items 67–69, addresses whether the caregiver would like to receive professional care and gets it. The sixth module, items 70–80, addresses the consequences of the patient’s illness for the patient’s children (if applicable). The seventh and last module is an open question intended to give the interviewee an opportunity to provide additional remarks. The question “Do you receive help/support in caring for the patient?” was added to the open question in this module. If the response to the “help” question is affirmative, the caregiver is then asked whether more help/support, the same amount help/support or less help/support was needed.

The reliability of the IEQ has been documented for different populations in several European countries showing not only good internal consistency for the subscales and the total score (van Wijngaarden 2003) but also sensitivity to change over time (Stam and Cuijpers 2001). The IEQ has been validated for the caregivers of patients with psychosis, patients with depression (van Wijngaarden 2003) and patients with brain damage (Geurtsen et al. 2010).

The Level of Expressed Emotion (LEE) (Gerlsma et al. 1992) is a questionnaire grounded in a prior study of expressed emotion (Vaughn and Leff 1981). The LEE assesses perceptions of expressed emotion on the part of the caregiver and is completed by both the patient and the caregiver. The questionnaire contains 38 items, which can be classified into four sub-scales (lack of emotional support, irritation, intrusiveness, criticism). All of the LEE items are scored along a 4-point Likert scale (1 = untrue, 2 = more or less untrue, 3 = more or less true, 4 = true). The time frame for the items is, just as for the IEQ, the prior 4 weeks. The total of the scores for the four sub-scales indicates the perceived extent of caregiver expressed emotion.

The LEE has been shown to have good internal consistency, reliability, construct validity and independence for gender, age and quantity of contact (Cole and Kazarian 1988; Gerlsma and Hale 1997; Nelis et al. 2010).

For the patients, the LEE was administered during a face-to-face interview. For the caregivers, both the LEE and the IEQ questionnaires were mailed to their home addresses. A telephone contact number accompanied the questionnaires for any information or other help which the caregivers might need.

### Statistical Analyses

Before testing our hypotheses, groups were compared on initial characteristics like the kind of relationship between care giver and patient and weekly contact hours, using chi2 test for significance for categorical data and ANOVA for data on interval/ratio level (*p* < .05).

For *research question 1,* the mean subscale scores and overall scores for the *core* module of the IEQ were calculated along with their standard deviations (SDs). Caregiver distress was determined on the basis of the number of items from the GHQ for which a score of 2 or 3 was assigned by the respondent (i.e., the answer differed negatively from the respondent’s “usual self”); caregivers responding with 3 or more of these scores were considered “distressed” while those responding with 2 or less were considered “not distressed”. A one-way ANOVA (*p* < .05) was then conducted to test for significant differences in the IEQ and LEE scores for the patient groups with the use of the Cohen’s *d* to guide interpretation when significant differences were found (*d* < 0.20 no relevant difference; *d* = 0.20–0.30 small difference; *d* = 0.30–0.80 moderate difference; and *d* > 0.80 large difference; Cohen 1988). The Chi^2^ statistic was used to test for significant differences between the percentages of distressed versus non-distressed caregivers according to the GHQ for the different groups of patients.

For *research question 2,* the four LEE sub-scale scores were summed to obtain a total expressed emotion score. The total LEE scores from the patient’s perspective and the caregiver’s perspective were then compared for each patient group. Just as for the IEQ scores, one-way ANOVAs (*p* < .05) were conducted to test for significant differences in the LEE scores across the patient groups with, once again, the Cohen’s *d* used to guide the interpretation of significant group differences (see above).

For *research question 3*, paired t-tests (*p* < .05) were used to evaluate the differences between the perspectives of the patients and the caregivers on expressed emotion for the three groups of patients. The Cohen’s *d* was (again) used to guide the interpretation of significant differences (see above).

For *research question 4*, the Pearson correlation coefficients between the sum scores for the IEQ and the LEE were calculated to examine the relation between expressed emotion and caregiver burden for the three groups of patients.

The three patient groups differed significantly in the average number of weekly contact hours with their caregivers. It was therefore decided to also conduct a series of post-hoc multivariate analyses to see if significant group differences in caregiver burden and expressed emotion *remained* after control for the amount of caregiver/patient contact on average.

## Results

### Patient Characteristics

Out of the 122 patients who participated in the Kronenberg et al. (2014) study, a total of 77 proved willing to complete the LEE. The other 45 patients were not interested in participating because they did not have a caregiver or did not want to bother their caregiver more than necessary. Of the 77 caregivers who were approached, 17 did not return both the IEQ and the LEE. This left a total of 60 patient/caregiver pairs for inclusion in the present study: 20 in each diagnostic group. The numbers being even was coincidental.

The intensity of care needed—according to the European Addiction Severity Index (EuropASI)—for the subgroup of 60 patients did not differ from the 122 patients included in the prior study with the exception of less gambling problems in the 20 SUD+ASD patients in the current sample than in the previous sample of 31 SUD+ASD patients.

### Caregiver Characteristics

Table 1 shows some 80 % of the patients to be male and some 80 % of caregivers to be female. The mean ages of the patients and their caregivers were comparable across the three groups of patients with the caregivers 5 years older than the patients on average.

**Table 1:** Demographic Characteristics of patients and their caregivers (*n* = 60)

	SUD		SUD+ASD		SUD+ADHD		Phi
	*N* = 20		*N* = 20		*N* = 20		
	Mean	sd	Mean	sd	Mean	sd	
Patient gender = female	20 %		10 %		30 %		0.287
Caregiver gender = female	75 %		85 %		85 %		0.641
Patient age	45.5	14.4	38.2	13	38.9	11.2	0.086
Caregiver age	51.4	14.7	45.7	15	46	13.6	0.376
Time of first mental health problems							0.244
<3 years	20 %		10 %		0		
3–10 years	10 %		15 %		15 %		
>10 years	45 %		45 %		60 %		
Unknown	25 %		30 %		25 %		
Relation caregiver to patient							0.365
Parent	20 %		30 %		30 %		
Child/sibling/family	20 %		30 %		15 %		
Partner	50 %		15 %		35 %		
Other	10 %		25 %		20 %		
Hours of contact per week							0.035*
<8	45 %		65 %		45 %		
09–32	5 %		5 %		30 %		
>32	50 %		20 %		25 %		
Missing	0		10 %		0		
Caregiver has children under 16	15 %		15 %		30 %		0.392

With the exception of the number of contact hours with the patients on average, the demographic characteristics of the caregivers were comparable across the three groups of patients. In the SUD group, 50 % of the caregivers reported more than 32 h of contact per week; this was true for only 20 and 25 % of the caregivers in the SUD+ADHD and SUD+ASD groups, respectively (*p* = 0.035).

### Caregiver Burden

Table 2 presents the IEQ core module scores for the three patient groups.

**Table 2:** Overview of caregiver burden and expressed emotion

Group		IEQ score range 0–118		GHQ distressed		LEE caregivers		LEE patients		
		Mean	sd	Caregivers percentage		Mean	sd	Mean	sd	
1	SUD *N* = 20	18.2	17.3		25 %	71.7	15.8	66.4	14.4	
2	SUD+ASD *N* = 20	13.1	9.1		55 %	64	18.1	68.9	17.4	
3	SUD+ADHD *N* = 20	16.3	13.1		45 %	67.2	18.5	64	19	
		IEQ		GHQ		LEE caregivers		LEE patients		LEE persp. CG < − > Pt
ANOVA (p-value)		0.59				0.573		0.513		
Paired *t*-test (p-value)										0.413
Chi^2^ (p-value)				0.209	*(df 3)*					
Cohen’s *d*										
Groups 1–2		0.364				0.453		0.156		0.351
Groups 2–3		0.284				0.174		−0.268		−0.276
Groups 1–3		−0.123				0.262		0.142		0.171

A moderate (*d* = .36) but non-significant difference was found between the total IEQ scores for the SUD group versus the SUD+ASD group. A small (*d* = .28) but non-significant difference was found for the SUD+ASD group versus the SUD+ADHD group. And no difference (*d* = −.12) was found in the total IEQ scores for the SUD group versus the SUD+ADHD group.

Although the caregivers for patients with SUD and a co-occurring ADHD or ASD both showed relatively high frequencies of being distressed compared to the caregivers for patients with only SUD (45 and 55 % versus 25 %), the differences in the GHQ scores for the groups were not significant (*p* = 0.209).

Inspection of Table 3 further shows no significant differences in caregiver burden across the three groups of patients. Financial problems for the caregiver due to patient problems did not differ across the groups of patients. The receipt of help and the need for additional help according to the caregivers also did not differ across the three patient groups.

**Table 3:** Further comparison of caregiver burden in terms of finances and help

	SUD *n* = 20	SUD+ASD *n* = 20	SUD+ADHD *n* = 20	Chi^2^ *p*-value
Financial consequences	45 %	25 %	45 %	0.714
Getting help	35 %	30 %	20 %	0.743
Wanting more help	25 %	5 %	10 %	0.341

The total number of caregivers with children under the age of 16 years was small (see Table 1). The responses of the caregivers to the sixth module of the IEQ, which addresses the consequences of the patient’s illness for the patient’s children, ranged widely and were therefore not analyzed further.

The open question in module 7 of the IEQ was responded to by 70 % of the caregivers, but the responses did not provide new information and were therefore not examined further.

### Expressed Emotion According to Caregivers and Patients

Additional inspection of Table 2 shows no significant differences in the perceptions of caregiver expressed emotion across the three groups of patients: No differences from the patient’s perspective and no differences from the caregiver’s perspective. The total LEE scores show no relevant differences in expressed emotion from the perspective of the caregiver for the SUD+ASD versus the SUD+ADHD groups or the SUD versus SUD+ADHD groups; the SUD group scored moderately (*d* = .45) but non-significantly higher than the SUD+ASD group for expressed emotion from the perspective of the caregiver. All of the groups showed similar amounts of caregiver expressed emotion when viewed from the perspective of the patient.

The perspectives of the patients and caregivers on expressed emotion of the caregiver also did not differ significantly when considered across the three patient groups) (*p* = 0.413).

### Effects of Number of Contact Hours

Given the differences in the number of contact hours between patient and caregiver across groups on average (*p* = 0.035) (see Table 1), multivariate analyses were performed to control for the possibly mediating influence of number of contact hours on caregiver burden and expressed emotion (Table 4).

**Table 4:** Effects of comorbid diagnosis and number of contact hours on burden

Variance explained by	IEO			GHQ			LEE caregivers			LEE patients		
	B	Std. err	Sig	B	Std. err	Sig	B	Std. err	Sig	B	Std. err	Sig
+ASD	−0.095	0.163	0.562	0.434	0.152	0.006	−4.965	5.391	0.361	5.653	5.321	0.294
+ADHD	−0.120	0.154	0.439	0.227	0.143	0.120	−4.164	5.121	0.420	−1.585	5.005	0.753
Hours of contact	0.071	0.036	0.051	0.106	0.033	0.002	3.881	1.203	0.002	3.259	1.161	0.007

The number of contact hours significantly affected all indicators of caregiver burden and expressed emotion. An additional diagnosis of ASD affected caregiver burden but only for “being distressed”: A diagnosis of a comorbid ASD was associated with a significantly higher level of caregiver distress.

### Caregiver Burden in Relation to Expressed Emotion of Caregivers

Table 5 shows both the LEE scores from the perspectives of the patients and the caregivers to correlate significantly with caregiver burden in general (i.e., the IEQ sum score).

**Table 5:** Correlations between caregiver burden as measured by the IEQ and caregiver expressed emotion as measured by the LEE from the perspective of the patients and from the perspective of the caregivers

	Sum score IEQ Pearson Sig. (2-tailed)	Sumscore SUD	Sumscore SUD+ASD	Sumscore SUD+ADHD
Sums core LEE pt	0.362**	0.288	0.302	0.599**
Sums core LEE cg	0.500**	0.644**	0.121	0.622**

For the specific groups of patients, significant correlations were found for the SUD group and the SUD+ ADHD group. Perceptions of expressed emotion from only the perspective of the caregivers correlated with caregiver burden for the SUD group. Expressed emotion from both the perspectives of the caregivers and the patients correlated with caregiver burden for the SUD+ADHD group.

No significant correlations between expressed emotion and caregiver burden were found for the SUD+ASD group.

## Discussion

In contrast to hypotheses 1 and 2, the co-occurrence of ADHD or ASD in addition to SUD was *not* significantly associated with greater caregiver burden or more expressed emotion when compared to the occurrence of SUD alone. Hypothesis 3, was also rejected: the patients’ scores for perceived caregiver expressed emotion did not differ significantly from the caregivers’ scores. Hypothesis 4 was confirmed : The LEE scores from the perspectives of both the patients and the caregivers significantly correlated with the IEQ sum scores for caregiver burden. The caregivers for patients with only SUD showed higher (although not significantly higher) levels of caregiver burden but also expressed emotion when compared to the caregivers for patients with SUD and a co-morbid ASD. This difference was found to be explained by the relatively higher number of weekly contact hours for particularly the SUD alone group.

More contact hours between caregiver and patient appear to be associated with higher levels of caregiver burden (IEQ sum scores), higher levels of perceived stress on the part of caregivers (GHQ distressed scores) and greater perceptions of expressed caregiver emotion (LEE scores from both the perspectives of the patients and the caregivers themselves).

The finding of a significant role for the amount of caregiver/patient contact is consistent with findings for the caregivers of patients with schizophrenia and dementia (Lauber et al. 2005; Scazufca and Kuipers 1998; Ulstein et al. 2007).

When the results for the IEQ in the present study are compared to the results of studies examining populations of patients with a bipolar disorder (Goossens et al. 2008) or schizophrenia (Van Wijngaarden 2003), the burden for the caregivers of patients with SUD—either with or without a co-morbid ASD or ADHD—is found to be *similar or even worse* than the burden for caregivers of patients with a bipolar disorder or schizophrenia.

The remaining question to be answered—of course—is just why the caregivers for patients with only SUD spend more time with their patients than the caregivers for patients with SUD and a co-morbid ADHD or ASD. A potential explanation is that the caregivers for patients with SUD and a co-morbid ADHD or ASD were already caring for these patients prior to the SUD (i.e., addiction). This care has perhaps occurred since childhood and the problems associated with the ADHD or ASD thus become a normal part of the caregivers life with strategies developed to deal with the burden of these problems and prevent stress—which may include *reducing* the intensity of the contact at times and the reduction of the total number of contact hours. For patients with only SUD, such a process is less likely to have taken place because the problems most likely started later in life.

A possible explanation for the caregivers of patients with only SUD showing higher levels of caregiver burden and expressed emotion is that patients with SUD often are subject to the social phenomenon that addiction is presumed to be due to a person being weak-willed rather than being ill, thus resulting in engagement with less compassion. This concept of disease considers SUD to be a patient’s choice instead of illness, and thus something which can be controlled by the patient him/herself. In contrast, a psychiatric disorder (in this case a co-morbid ADHD or ASD) is considered fate/illness, and thus something which cannot be controlled. This social conception of disease can presumably influence scores on the IEQ, GHQ and LEE: *increase* scores for patients with only SUD and *decrease* scores for patients with SUD and a co-morbid ADHD or ASD. A similar effect has been reported in the work of Kronenberg et al. (2014) where SUD+ADHD and SUD+ASD patients reported that their caregivers became *more involved* and *less reproachful* once they were also diagnosed with ADHD or ASD. This is also a possible explanation for the finding in the present study of no significant correlations between caregiver burden (IEQ) and perceptions of expressed emotion (LEE) for the SUD+ASD group.

Finally, it is certainly possible that SUD patients may just be more “claiming”: the substance use is persistently demanding the attention in their relationships with caregivers. This explanation is supported by: the higher perceptions of expressed emotion for the caregivers in the SUD group; the strong correlations between the IEQ scores (caregiver burden) and LEE scores (expressed emotion) for this group; and the high GHQ scores (distressed caregivers) for this group.

### Strengths and Limitations of the Present Study

To our knowledge, this is the first study to compare caregiver burden in SUD patients with and without co-morbid ADHD or ASD. The findings must nevertheless be interpreted with caution for a number of reasons. To start with, only 60 of the 122 patients we approached had caregivers who were willing to participate in the present study. When the clinical profiles of the participants were compared to those for the total group of 122 patients, the profiles were found to be very similar, which suggests that the external validity of the current findings is likely to be good. The generalizability of the conclusions with regard to other caregivers needs to be checked, however. Second, the sample size was limited. The power of the study was therefore restricted, and the possibility of type II errors cannot be excluded. In order to address this risk, we used a lenient threshold for statistical significance (*p* < 0.10) and did not correct for multiple testing. This means that the findings of the present study must be replicated using another, larger sample. Third, the data were obtained using self-report questionnaires. When replicating the study in future research, it is therefore recommended that in-depth interviews also be conducted with informal caregivers. Also, it is recommended to conduct qualitative research using grounded theory. The results of a grounded theory project could allow to build a richer narrative that would yield a constructive, conceptual framework that would allow for the generation of further more targeted hypotheses.

Finally, the design of the present study was cross sectional. Causal conclusion therefore cannot be drawn and longitudinal research might therefore be undertaken in the future.

### Clinical Implications

Considering the importance of informal caregivers as partners in the care for patients with psychiatric disorders and the relatively high burden of caring for patients with SUD and possibly co-morbid ADHD or ASD, clinicians should take this burden into account. The results of this study show that the number of contact hours between patient and caregiver may be a simple and straightforward indicator of caregiver burden. Assessment of expressed emotion and the number of weekly contact hours with informal caregivers can therefore be recommended to quickly identify caregivers who are vulnerable and thus at a high risk for psychological problems themselves. The LEE is easy to administer and, according to the results of this study, the presence of the informal caregiver is not necessarily needed; administration to the patient may provide accurate and sufficient information. In the future, the expressed emotion of caregivers may be decreased with the use of motivational interviewing and the training of interaction skills to improve their strategies (Smeerdijk et al. 2012). The number of contact hours with a patient might also be decreased for individual caregivers and/or the number of informal caregivers increased and, in such a manner, the burden on caregivers for the mentally ill be kept to a minimum.
